# Comparative Study of Attitudes towards Communication Skills Learning between Medical and Dental Students in Saudi Arabia

**DOI:** 10.3390/ijerph18010128

**Published:** 2020-12-27

**Authors:** Ahmed Abed Elwahab Nourein, Rubayyi Faris Shahadah, Marwan Abdulrahman Alnemer, Saif Saud Al-Harbi, Hani T. Fadel, Saba Kassim

**Affiliations:** 1 Department of Oral and Maxillofacial Surgery, College of Dentistry, Taibah University, AlMadinah AlMunawwarah 42313, Saudi Arabia; nourein2@gmail.com; 2 College of Dentistry, Taibah University, AlMadinah AlMunawwarah 42313, Saudi Arabia; rabeafs20@gmail.com (R.F.S.); marwanalnemer@gmail.com (M.A.A.); Saif.saud417@gmail.com (S.S.A.-H.); 3 Department of Preventive Dental Sciences, College of Dentistry, Taibah University, AlMadinah AlMunawwarah 42353, Saudi Arabia; drhfadel@yahoo.com

**Keywords:** attitudes, communication skills, medical, dental, students, traditional, problem-based learning

## Abstract

*Background*: Communication skills (CS) learning is a core skill in medical and dental education. The comparison of attitudes towards CS between dental and medical students based on the taught curriculum (problem-based learning vs. traditional teaching) in Saudi Arabia awaits investigation. Aims: (1) To assess the attitudes of both undergraduate dental and medical students towards communication skills (CS) learning and (2) to compare the attitudes towards CS between Medical and Dental students in relation to sociodemographic and education-related characteristics. *Methods and Materials*: A cross-sectional study, using an online survey, invited 260 conveniently sampled Taibah university medical and dental undergraduate students. The survey collected data on sociodemographic characteristics, education-related factors, and CS using Communication Skills Attitude Scale (CSAS) that assess positive and negative attitudes (PAS, NAS). Data analysis included descriptive statistics and the Mann–Whitney *U* test. *Results*: Of the distributed questionnaire 91% responded (145 dental and 91 medical students). There were, overall, non-significant scores’ differences between medical and dental students on PAS (Medicine Median 51 vs. Dentistry Median 50, *p* = 0.059) and NAS (Medicine Median *32* vs. Dentistry Median 32, *p* = 0.596). Older medical students, those at clinical levels and those who reported they need to improve their communication skills and student whose parents were not doctors, tended to score statistically significantly (*p* = 0.032, 0.017, 0.034, and 0.004, respectively) on PAS compared with dental students; on the other hand, medical students with doctor parents scored significantly high in NAS compared to dental students (*p* = 0.015). *Conclusion*: Demographic and education-related characteristics underpinned medical student positive attitude towards CS compared to dental students. Although medical and dental students showed no differences in self-rating their attitudes towards (CS). Different factors influence medical and dental students’ attitudes towards CS learning.

## 1. Introduction

Communication by health care professionals is a key process in a patient–clinician relationship [1]. Communication skills (CS) learning is an integral component of medical and dental education, these core skills positively contribute to various aspects of the healthcare process, including conducting a meticulous examination, reaching a correct diagnosis, formulating a comprehensive plan, and providing adequate treatment [2,3]. Recent initiatives from authoritative medical educational bodies, licensing examination formats, and accreditation procedures are probing these essential core skills. Notably, medical educational authorities recommend that medical and dental graduates should have certain levels of mastery of CS for interactions with patients, colleagues, and team members; medical and dental schools are required to tailor their undergraduate curricula as such to incorporate adequate CS learning and training [4]. Medical and dental schools in Norway, Belgium, Switzerland, and Malaysia have implemented CS learning and training at different education levels within their programs [5,6,7,8,9]. Several sociodemographic and education factors were reported to influence positive and negative students’ attitudes towards CS learning. These included age [10,11], gender [11,12,13], students’ self-rating CS [14,15], need of improvement [11], doctor parents, being socialized to negative attitudes [10,11,12,13,14,15], ethnicity and cultural beliefs [11], course contents [11], timing of the course offered [13], teaching methods [13], learning environment [16], assessment methods [11,17], apathy to CS [18], image as ‘social non-clinical science’ by medical students [19,20], and school curriculum type [7,21,22].

Evidence showed that there is a gap of knowledge among medical graduates in terms of necessary soft or supporting skills [23,24]. Studies have also demonstrated that communication skills are teachable and trainable [13,17,25]. Since the student is the primary customer or stakeholder in the educational process, a student-centered curriculum should address the students’ needs, values, learning experiences, behaviour, and beliefs. As communication is an integral part of health sciences education, exploring this area from a behavioral aspect is of direct practical importance for policymakers. Moreover, students’ attitudes towards communication skills impacts the overall time spent for learning [10]. However, the attitude of the undergraduate students to uptake the CS skills could be predicted by theory of planned behaviour (TPB), that suggests behaviour can be predicted by intention to engage in behaviour. Research on students’ attitudes towards effective communication skills may ultimately influence the used learning strategies in health sciences programs [26].

In Saudi Arabia (SA), a significant increase in the number of dental and medical colleges in the last two decades has been observed [27]. This was accompanied by a reform in medical education, including the adoption of the ‘Saudi Meds’ framework, among the domains of which clearly listed were CS [28]. The competencies of these CS include demonstrating appropriate CS and behavior with patients, their families, colleagues, other health professionals, and the public, applying the general principles of CS, using different methods of communication with different patients at different situations and breaking bad news [23,28].

In the health sciences education literature in general, several studies have compared communication skills among traditional and problem-based curricula. A cross-sectional survey on four different universities showed that students at traditional curriculum scored the lowest at knowledge acquisition in communication skills when compared to students of two PBL schools and students of one integrated medical school [22]. In another nationwide study in Norway, a clear difference was observed on how communication skills were reported between the traditional curriculum students and those among integrated curricula students [21].

Current literature suggests that there is a paucity of research in SA, comparing medical and dental (or traditional and integrated) students’ attitudes toward CS learning and training. At Taibah University in Madinah, SA, the College of Medicine (COM) has adopted a reformed problem-based learning (PBL) undergraduate curriculum, that integrates all levels of the program. The College of Dentistry (COD), on the other hand, still utilizes a traditional approach that arguably lacks CS as a main component in the curriculum. Comparing the two colleges in terms of student attitudes towards CS learning and training would provide a unique opportunity to study the influence of both learning approaches i.e., traditional vs. integrated curricula, and identify possible determining or contributory factors. Thus, the aims of this study were [1] to assess the attitudes of both undergraduate dental and medical students at Taibah University towards communication skills learning and [2] to compare the attitudes towards communication skills between medical and dental students in relation to sociodemographic and education-related characteristics.

## 2. Materials and Methods 

### 2.1. Study Design, Setting and Sampling

This analytical cross-sectional study recruited a convenience sample of undergraduate students attending colleges of Medicine and Dentistry at Taibah university in Madinah, Saudi Arabia, during the academic year 2019–2020. Students enrolled in the common preparatory year, internship program and postgraduate residency training were excluded. The inclusion of undergraduate medical and dental students was on the basis that by the end of the undergraduate program students should have acquired and demonstrated proficiency in communication skills (CS) and are able to provide advice and explanation to patients and their families and relatives [11].

A cluster sampling was undertaken from the total number of medical and dental students. Each student representative in every academic level distributed the questionnaire to his or her group, and responses from the first 20 participants were deemed adequate. In this study the strategy of sample size calculation was based on the statistical validation of the Communication Skills Attitude Scale (CSAS), i.e., item-subject ratio and was within the range of 1:20, as the literature suggests [29]. Accordingly, the required sample size calculated was 236 and increased to 260 to compensate for non-respondents.

### 2.2. Measurements, Data Collection and Ethical Approval

An English self-administered questionnaire (Supplementary Material) was used to assess students’ attitudes towards communication skills. This questionnaire is composed of two sections: section one asked questions about age, gender, college, academic year, self-reported socioeconomic status, job of parents (medical doctors or other), self-rating of communication skill, and whether students thought their communication skills needed improving, the latter three questions were adapted from relevant literature [13,14]. 

Section two focused on communication skills using the Communication Skills Attitude Scale (CSAS) [17,18]. The CSAS is a validated, well-established instrument used for assessing CS [18]. The CSAS was transculturally adapted into different languages, i.e., German, Korean, and Norwegian, and was used as a probing tool for students’ attitudes towards CS learning [10,30,31]. The selection of the CSAS scale allows comparison of findings of the present study with other relevant studies using the same scale. This scale consists of 26 items: 13 positive (CSAS–PAS) and 13 negative statements (CSAS–NAS) [10]. The items are anchored to a five-point Likert-scale responses, ranging from 1 (strongly disagree) to 5 (strongly agree) and these items were presented randomly in the scale [10]. 

The questionnaire was preceded with cover page that stated the aims of the study, anonymity and confidentiality of the responses and the voluntarism of the participation. In addition, the ethical committee that approved the study and the contact details of one the team investigators for any queries was made available. It took approximately ten minutes to fill and was sent electronically via a WhatsApp message that linked to a Google form. The responding students were instructed on completion filling the form, to submit the web form to the web server. This latter inserted the collected data into Excel spread sheet that stored the data for retrieval and analysis. The Taibah University College of Dentistry Research Ethics Committee reviewed and approved the study (Ref: TUCDREC/20200322/AANourein). The study adhered to the World Medical Association guidelines set at the Declaration of Helsinki. The questionnaire responses were anonymous and were coded during data analysis. The participants’ informed consent was waived as the research ethics committee approved the study and deemed it as self-administered questionnaire with voluntary and anonymous participation. In addition, no personal information would be obtained. However, the submission of the questionnaire was considered as a proxy of consent to participate in the study. 

### 2.3. Statistical Analysis 

The Statistical Package for Social Sciences 16 (SPSS, version 16, Inc., Chicago, IL, USA) was used for data analysis. Previous relevant studies guided the statistical analysis plan for the present study [10,32]. Descriptive analysis was performed to summarize sample characteristics and were presented as median and interquartile range (IQR), as all the continuous data did not adhere to normality distribution (Kolmogorov–Smirnov ≤ 0.05), and frequencies with percentages (F%) for categorical data. The overall internal consistency of the CSAS was tested alongside the subscales CSAS–PAS and CSAS–NAS, for medical and dental students, using Cronbach’s alpha coefficient (α). The statistically significant differences for the CSAS–PAS and CSAS–NAS between the medical and dental students in relationship to sociodemographic and education-related characteristics was determined using the Mann–Whitney *U* test. The number of variables in Table 1, result section was recategorized. The socio-economic status (SES) was recategorized to ‘Other levels [low and middle]’ and ‘High level’ due to small number [33] of students with low SES. In addition, the self-reported communication skill was recategorized into ‘Excellent’, ‘Very good’, ‘Fair’ and ‘Poor’ and Very poor’ to have meaningful analysis. The age of students was categorized according to training level. The significance level was set at *p*-value ≤ 0.05.

## 3. Results

### 3.1. Total Sample Characteristics and Internal Consistency of the CSAS

Table 1 shows the whole set of sample characteristics, and attendance at the according college (Medical and Dental). The median (IQR) age of the whole participating students was 22.0 [3] years, 91 (36.6%), and 145 (61.4) of the responding students were from medical and dentistry college. The majority (70%) of the responding students were at clinical levels. The overall internal consistency (α) of the CSAS and for the subscales (PAS and NAS) was 0.70, 0.88, and 0.63, respectively. As for the medical students, internal consistency (α) of the CSAS and for the subscales (PAS and NAS) was 0.56, 0.85, and 0.55, respectively; meanwhile the α of the CSAS among dental students was 0.75 and for the subscales (PAS and NAS) 0.90 and 0.67, respectively. 

### 3.2. Comparisons of Attitudes towards CS Skills between Medical and Dental Students 

There was over all non-significant scores’ differences between medical and dental students on PAS (Medicine Median 51 vs. Dentistry Median 50, *p* = 0.059) and NAS (Medicine Median 32 vs. Dentistry Median 32, *p* = 0.596). However, as shown in Table 2, older medical students, those at clinical levels, those who self-reported that they need to improved their communication skills and that their parents were not doctors, tended to score statistically significantly (*p* = 0.032, 0.017, 0.034, 0.004, respectively) on PAS compared with dental students. Surprisingly, medical students who reported their parents’ job as doctors scored statistically significantly on NAS compared to counterparts’ dental students (*p* = 0.015).

## 4. Discussion

The current investigation aimed at assessing the attitudes of both undergraduate dental and medical students towards communication skills learning. To the best of our knowledge, this is the first study in Saudi Arabia that carried out such an attempt. In this study, although positive (PAS) and negative (NAS) attitudes of medical and dental students towards communication skills (CS) learning were generally similar, both are influenced by different factors; medical students’ (PAS) positive attitude scale towards communication skills learning were related to older age, clinical level training students, low self-rated students’ views on communication skills, and non-medical family background. The (PAS) of dental students, on the other hand, were more related to high student self-rating in communication skills. It is worth mentioning that the (NAS) negative attitudes scale towards communication skills learning of medical students were related to medical family background (parent-doctors). 

The findings of this study, similarity of PAS and NAS among medical and dental students agreed with those reported by Lumma–Sellenthin who stated that preliminary disillusional results showed no general superiority of PBL over traditional teaching methods, while detailed studies found differences in certain areas [34]; the researcher supported her statement with evidence from the literature [35,36,37,38]. An explanation could be related to the cross-section study type, although cross-sectional studies provide an exploratory approach; evidence conclude that longitudinal studies are required for such investigation [34]. Another reason is that most of the respondents (70%) in this study were clinical students from both colleges; this attributes to their reflection on being taught within the clinical environment that foster (CS) valuing and practicing.

The present study also aimed at comparing the attitudes towards communication skills between medical and dental students in relation to sociodemographic and education-related characteristics. Among medical students, older students (≥22 years of age), who happened to be at the clinical level of training, were scoring significantly higher in PAS in comparison to their dental students’ counterparts. This finding is attributed to the fact that students at higher levels of a PBL program in medical school are exposed to higher cognitive and advanced learning, i.e., Phase 2: ‘Developing clinical competencies’ and Phase 3: ‘Preparation for practice’ and ‘Consolidation of information’, in comparison to their dental counterpart at traditional lectured-based curriculum. It was mentioned earlier in this paper that the literature showed clear differences between PBL students in comparison to tradition curriculum students in how communication skills are reported [21]; additionally, the traditional students scored the lowest in communication skills in comparison to PBL and integrated-curriculum students [22]. 

In SA, at Altaif University College of Medicine, paradoxically, Alotaibi et al. [39] observed that older students showed significantly higher PAS and NAS attitudes towards communication skills learning compared to younger students [39].

It is worth mentioning that this study was held at the end of the academic year, no communication skills course per se was conducted; rather, communication skills training and assessment sessions were incorporated within the curricular activities, while the main theme of the previous studies was investigating students’ attitudes towards communication skills learning at the event of a recently taught communication skills course [14,30,40].

The current study showed that medical students who self-rated themselves as ‘Poor’ in their communication skills scored significantly high in PAS, while scoring was significantly high in NAS for students who self-rated themselves as ‘Very Good’ and ‘Excellent’. This finding is consistent with the previous studies, as the evidence showed that students who self-rated themselves badly score high in their PAS [11]. 

As for dental students, those who self-rated themselves as ‘Very Good’ and ‘Excellent’ communicators showed positive attitudes towards communication skills learning. Although this is inconsistent with the previous studies on medical students [11,14], some studies reported this finding [41,42]. In the dental education literature, however, Nor Nor et al. [9] found that dental students who rated themselves as ‘Poor’ communicators showed negative attitudes towards communication skills learning. Nevertheless, the literature also reported that students’ self-rating and self-efficacy in communication skills encouraged medical educators to provide more engaging student-centered instructional methods [6]. Interestingly, neither medical college nor dental college showed significant differences in attitudes towards (CS) were observed between males and females, a finding inconsistent with the mainstream of the literature [10,12,41,43]. However, similar findings were obtained from a study in Sri Lanka [44].

The present study did not find a significant relationship between the self-reported socioeconomic status of medical students and their attitudes towards communication skills learning. Similarly, the self-reported socioeconomic status of dental students was non-significant in relation to their attitudes towards communication skills learning. No study showed a correlation between the self-perceived socioeconomic status of dental students with regards to their attitudes towards communication skills learning. In the literature, some studies defined socioeconomic status as whether the students had any working experience in health services [10,45]. While in the current study, socioeconomic status referred to the self-perception of such status by the students. As Taibah University is a governmental university, all students are granted free education, consequently aiding their dedication to learning.

The current study also showed that NAS among medical students towards CS learning were associated with parents of medical backgrounds, i.e., physicians. This finding is similar to other studies [11,45] that hypothesized that there was a possible transformation of poor attitudes from experienced practicing health care professionals, i.e., ‘doctor parent’, to immature ‘student siblings’, since communication skills at the time of the parents’ education were not taught as of today’s teaching [11].

One limitation that should be acknowledged is that this study was a cross-sectional in nature, thus causality is precluded. Second, the findings were reported from one institute in Saudi Arabia. Therefore, generalizability of the findings to dental and medical institutes within the context of SA is not possible. Moreover, the self-selection of the students within the institute to participate in the study could have biased the results. One should consider that this study was conducted during the pandemic of COVID-19, i.e., students were under urgent re-arrangement of their environment of education, which might have made them deprioritize the participation in the survey. Finally, the self-reporting of CSAS could have invoked social desirability, therefore, self-reporting of CSAS would have been enhanced and validated if objective measures were utilized.

## 5. Conclusions

Within the limitations of this study, it can be concluded that positive (PAS) and negative (NAS) attitudes of medical and dental students towards communication skills (CS) learning were generally similar. Medical students’ (PAS) towards communication skills learning were related to older age, clinical level training students, non-medical family background and low self-rated students’ views on communication skills. The (PAS) of dental students, on the other hand, were more related to high self-rating in communication. Parents with medical backgrounds were associated with NAS towards CS learning more among medical students than in dental students. Further studies are needed to provide educational leaders with evidence-based feedback for improvement of communication skills learning courses, to probe for feedback weaknesses, and for maximizing students’ potential towards more professional and patient-centered care.

## Figures and Tables

**Table 1:** Whole sample sociodemographic and education-related characteristics of participating students (236) and per college (Medical [*n* = 91], Dental [*n* = 145]).

Variable	Total Sample	Medical	Dental
	F (%) or M(IQR)	F (%) or M (IQR)	F (%) or M (IQR)
**Age/years**	22.0 (3)	21.0 (3)	23.2 (2)
**Gender**			
Male	129 (54.7)	45 (49.5)	84 (57.9)
Female	107(45.3)	46 (50.5)	61 (42.1)
**Self-reported socio-economic status**			
Low	3 (1.3)	1 (1.1)	2 (1.4)
Middle	220 (84.7)	74 (81.3)	126 (86.9)
High	33 (14.0)	16 (17.6)	17 (11.7)
**Training**			
Non-clinical (second and third year)	70 (29.7)	42 (46.2)	28 (19.3)
Clinical (fourth, fifth and sixth year)	166 (70.3)	49 (53.8)	117 (80.7)
**Self-rated of communication skill**			
Excellent	6 (2.5)	1 (1.1)	5 (3.4)
Very good	24 (10.2)	6 (6.6)	18 (12.4)
Fair	98 (41.5)	41 (45.1)	57 (39.3)
Poor	83 (35.2)	33 (36.3)	50 (34.5)
Very poor	25 (10.6)	10 (11.0)	15 (10.3)
Self-reported of needs to improve CS *			
Yes	211 (89.4)	82 (90.1)	129 (89.0)
No	25 (10.6)	9 (9.9)	16 (11.0)
**Father or mother or both doctors ^$^**			
Yes	27 (11.4)	11 (12.1)	16 (11.0)
No	208 (88.1)	80 (87.9)	128 (88.3)

* CS = communication skills; ^$^ = missing data for one participant; M(IQR) = Median with interquartile range.

**Table 2:** Comparisons of medical and dental students’ attitudes towards CS skills (*n* = 236).

Variable	PAS M(IQR)		*p*-Value	NAS M(IQR)		*p*-Value
	Medical	Dental		Medical	Dental	
**Age**						
≤21 years	49.0 (8.5)	48.0 (9.5)	0.188	23.50 (5)	34.0 (10)	0.758
≥22 years	53.0 (10)	50.0 (14.50)	0.032	31.0 (4)	32.0 (8)	0.177
**Gender**						
Male	51.0 (9)	50.0 (15.5)	0.200	32.0 (4)	32.0 (9)	0.953
Female	50.0 (9.25)	49.0 (13)	0.118	32.0 (4)	33.0 (8)	0.401
**Self-reported socio-economic status**						
Other	51.0 (8)	50.0 (14)	0.132	32.0 (5)	32.0 (8)	0.440
High	53.0 (10)	49.0 (6.50)	0.309	31.50 (11)	28.0 (12)	0.557
**Training**						
Non-clinical (second and third year)	48.50 (8.25)	48.0 (9.50)	0.275	32.0 (6)	34.0 (10)	0.885
Clinical (fourth, fifth and sixth year)	53.0 (9)	50.0 (14)	0.017	31.0 (4)	32.0 (8)	0.165
**Self-rated of communication skill**						
Excellent, Very good and Fair	45.0 (18)	42.0 (13)	0.666	36.0(10)	32.0 (9)	0.131
Very poor and poor	51.0 (9.75)	51.0 (12)	0.191	32.0 (5)	32.0 (9)	0.394
Self-reported of needs to improve CS *						
Yes	51.0 (9)	49.0 (13)	0.034	32.0 (4)	32.0 (8)	0.721
No	57.0 (17.50)	51.0 (10)	1.000	27.0 (10)	28.50 (12)	0.357
**Father or mother or both doctors**						
Yes	49.0 (8)	51.0 (7.75)	0.827	36.0 (9)	29.50 (7)	0.015
No	51 (10.75)	49.0 (14)	0.044	32.0 (5)	32.0 (10)	0.143

* CS = communication skills; M(IQR) = Median with interquartile range.

## Supplementary Materials

The Survey questionnaire is available online at https://www.mdpi.com/1660-4601/18/1/128/s1.
