# Supplementary material for: Comparative Study of Attitudes towards Communication Skills Learning between Medical and Dental Students in Saudi Arabia
Source: Int J Environ Res Public Health. 2020 Dec 27;18(1):128. doi: 10.3390/ijerph18010128 (PMC7795169; doi:10.3390/ijerph18010128)
Supplement: Supplementary file 1 [file ijerph-18-00128-s001.pdf]

Comparative Study of Attitudes Towards Communication Skills Learning between Medical and Dental Students in Saudi Arabia

We are a group of dental researchers based at the College of Dentistry, Taibah University (TU) Al Madinah AlMunawwarah, Saudi Arabia. The aims of this study are: (1) To assess the attitudes of both undergraduate dental and medical students towards communication skills (CS) Learning and (2) to compare the attitudes towards CS between medical and dental students in relation to sociodemographic and education-related characteristics.

*Your data will be dealt with in a high confidentiality and for scientific purposes only.

*The number of questions will be 34 , and the estimated time for completion is 10 minutes.

*The survey is anonymous and participation is voluntary.

Your participation is highly appreciated. If you have any comments/questions regarding the questionnaire, feel free to contact us at: nourein2@gmail.com or: +966505539489.

The Taibah University College of Dentistry Research Ethics Committee (TUCDREC) reviewed and approved the study (Ref: TUCDREC/20200322/AANourein)

*Dear participant, please be noticed that this questionnaire is available online at:

(https://forms.gle/nTsZhoRSapYP98cY7)

* Do you agree to participate in this study?

☐ Yes ☐ No

A. Personal Data & Education-related Characteristics

1) Gender:
- Male
- Female

2) College:
- College of Medicine
- College of Dentistry

3) Academic Year:
- 2nd Year
- 3rd Year
- 4th Year
- 5th Year
- 6th Year

4) Age: ☐

5) Socio-economic status:
- Low
- Middle
- High

6) Is your father or mother or both a doctor?
- Yes
- No

7) How do you rate your communication skills?
- 1
- 2
- 3
- 4
- 5

8) Do you think your communication skills need improving?
- Yes
- No

B. Communication Skills Attitudes Scale (CSAS)

Dear highly esteemed student, feel free to choose among the numbered points (1-5) that reflects your personal judgment.

Please read the following statements about communication skills learning. Indicate whether you agree or disagree with all the statements by circling the most appropriate response.

Remember, 1. strongly disagree 2. disagree 3. neutral 4. agree 5. strongly agree

9) In order to be a good doctor, I must have good communication skills:

- 1
- 2
- 3
- 4
- 5

10) I can't see the point in learning communication skills:

- 1
- 2
- 3
- 4
- 5

11) Nobody is going to fail their medical degree for having poor communication skills:

- 1
- 2
- 3
- 4
- 5

12) Developing my communication skills is just as important as developing my knowledge of medicine:

- 1
- 2
- 3
- 4
- 5

13) Learning communication skills has helped or will help me respect patients:

- 1
- 2
- 3
- 4
- 5

14) I haven't got time to learn communication skills:

- 1
- 2
- 3
- 4
- 5

15) Learning communication skills is interesting:

- 1
- 2
- 3
- 4
- 5

16) I can't be bothered to turn up to sessions on communication skills:

- 1
- 2
- 3
- 4
- 5

17) Learning communication skills has helped or will help facilitate my team-working skills:

- 1
- 2
- 3
- 4
- 5

18) Learning communication skills has improved my ability to communicate with patients:

- 1
- 2
- 3
- 4
- 5

19) Communication skills teaching states the obvious and then complicates it:

- 1
- 2
- 3
- 4
- 5

20) Learning communication skills is fun:
- 1
- 2
- 3
- 4
- 5

21) Learning communication skills is too easy:
- 1
- 2
- 3
- 4
- 5

22) Learning communication skills has helped or will help me respect my colleagues:
- 1
- 2
- 3
- 4
- 5

23) I find it difficult to trust information about communication skills given to me by non-clinical lecturers:
- 1
- 2
- 3
- 4
- 5

24) Learning communication skills has helped or will help me recognize patients' rights regarding confidentiality and informed consent:
- 1
- 2
- 3
- 4
- 5

25) Communication skills teaching would have a better image if it sounded more like a science subject:
- 1
- 2
- 3
- 4
- 5

26) When applying for medicine, I thought it was a really good idea to learn communication skills:

- 1
- 2
- 3
- 4
- 5

27) I don't need good communication skills to be a doctor:

- 1
- 2
- 3
- 4
- 5

28) I find it hard to admit to having some problems with my communication skills:

- 1
- 2
- 3
- 4
- 5

29) I think it's really useful learning communication skills on the medical degree:

- 1
- 2
- 3
- 4
- 5

30) My ability to pass exams will get me through medical school rather than my ability to communicate:

- 1
- 2
- 3
- 4
- 5

31) Learning communication skills is applicable to learning medicine:

- 1
- 2
- 3
- 4
- 5

32) I find it difficult to take communication skills learning seriously:

- 1
- 2
- 3
- 4
- 5

33) Learning communication skills is important because my ability to communicate is a lifelong skill:

- 1
- 2
- 3
- 4
- 5

34) Communication skills learning should be left to psychology students, not medical students:

- 1
- 2
- 3
- 4
- 5

Any other information/comments want to add?

………

………

This is the end of the questionnaire, thanks for your participation
